# Effects and mechanisms of 6-hydroxykaempferol 3,6-*di*-*O*-glucoside-7-*O*-glucuronide from Safflower on endothelial injury *in vitro* and on thrombosis *in vivo*

**DOI:** 10.3389/fphar.2022.974216

**Published:** 2022-09-13

**Authors:** Li-Wei Wang, Jiang-Feng He, Hai-Yan Xu, Peng-Fei Zhao, Jie Zhao, Cong-Cong Zhuang, Jian-Nan Ma, Chao-Mei Ma, Yong-Bin Liu

**Affiliations:** ^1^ State Key Laboratory of Reproductive Regulation and Breeding of Grassland Livestock, School of Life Sciences, Inner Mongolia University, Hohhot, China; ^2^ Key Laboratory of Herbage and Endemic Crop Biology of Ministry of Education, School of Life Sciences, Inner Mongolia University, Hohhot, China; ^3^ Biotechnology Research Institute, Inner Mongolia Academy of Agricultural and Animal Husbandry Sciences, Hohhot, China; ^4^ Center of Reproductive Medicine, The Affiliated Hospital of Inner Mongolia Medical University, Hohhot, China; ^5^ Department of Traditional Chinese Medicine Resources and Development, College of Pharmacy, Inner Mongolia Medical University, Hohhot, China

**Keywords:** *Carthamus tinctorius*, 6-hydroxykaempferol 3,6-*di*-*O*-glucoside-7-*O*-glucuronide (HGG), human umbilical vein endothelial cells (HUVECs), oxygen glucose deprivation/reoxygenation (OGD/R), phenylhydrazine (PHZ), anti-thrombosis

## Abstract

**Background:** The florets of *Carthamus tinctorius* L. (Safflower) is an important traditional medicine for promoting blood circulation and removing blood stasis. However, its bioactive compounds and mechanism of action need further clarification.

**Objective:** This study aims to investigate the effect and possible mechanism of 6-hydroxykaempferol 3,6-*di*-*O*-glucoside-7-*O*-glucuronide (HGG) from Safflower on endothelial injury *in vitro*, and to verify its anti-thrombotic activity *in vivo*.

**Methods:** The endothelial injury on human umbilical vein endothelial cells (HUVECs) was induced by oxygen-glucose deprivation followed by reoxygenation (OGD/R). The effect of HGG on the proliferation of HUVECs under OGD/R was evaluated by MTT, LDH release, Hoechst-33342 staining, and Annexin V-FITC apoptosis assay. RNA-seq, RT-qPCR, Enzyme-linked immunosorbent assay and Western blot experiments were performed to uncover the molecular mechanism. The anti-thrombotic effect of HGG *in vivo* was evaluated using phenylhydrazine (PHZ)-induced zebrafish thrombosis model.

**Results:** HGG significantly protected OGD/R induced endothelial injury, and decreased HUVECs apoptosis by regulating expressions of hypoxia inducible factor-1 alpha (HIF-1α) and nuclear factor kappa B (NF-κB) at both transcriptome and protein levels. Moreover, HGG reversed the mRNA expression of pro-inflammatory cytokines including *IL-1β*, *IL-6*, and *TNF-α*, and reduced the release of IL-6 after OGD/R. In addition, HGG exhibited protective effects against PHZ-induced zebrafish thrombosis and improved blood circulation.

**Conclusion:** HGG regulates the expression of HIF-1α and NF-κB, protects OGD/R induced endothelial dysfunction *in vitro* and has anti-thrombotic activity in PHZ-induced thrombosis *in vivo*.

## Introduction

Globally, coronary artery disease, particularly acute myocardial infarction (AMI) is one of the dominant causes of death and disability (Wang et al., 2020). AMI occurs when coronary artery becomes partially or totally occluded, leading to an acute loss of vital oxygen and glucose to heart tissue, thereby causing endothelial cells and tissue damage (Sattler et al., 2019). AMI is the major complication of atherosclerosis, thrombosis, etc., (Gulati et al., 2020). In AMI, due to the blockage of blood supply to heart, oxygen and nutrient supply restricts and cell apoptosis occurs, which triggers the intercellular signaling cascade. AMI occurs with the intense-inflammatory response to repair myocardial tissues, but exaggerated inflammatory response results in left-ventricular-remodeling and heart failure (Montrief et al., 2019). Vascular endothelial cells, uniquely positioned at the interface between the blood and tissue, plays a critical role in maintaining cardiovascular homeostasis (Wei et al., 2020). The injury or death of endothelial cells is believed to be the initial event in endothelial pathophysiological state, such as inflammation, vascular injury, and thrombosis (Qi et al., 2017; Mathiesen et al., 2021). In order to prevent acute myocardial infarction, it is necessary to relieve endothelial injury and eliminate thrombosis caused by ischemia and hypoxia.

Safflower is a traditional medicine renowned for its efficacy on promoting blood circulation and removing blood stasis (Chinese Pharmacopoeia Committee, 2020). Xuebijing (blood-cleaning) injection with Safflower extract as a major component could block Covid-19 proliferation, and inhibit the expression of pro-inflammatory cytokines (He et al., 2021). In addition, Xuebijing injection effectively improves cardiac function after hypoxia/reoxygenation injury (He et al., 2016). Safflower contains large amounts of chalcone and flavanols represented by hydroxysafflor yellow A (HSYA) and 6-hydroxykaempferol glycosides, respectively. Among them, HSYA has been the interest of Safflower bioactivity investigation in most documents, and its effects to protect endothelial cells against hypoxia-induced injury was also reported (Ji et al., 2009). However, little is known about the related effects and the underlying mechanism of the 6-hydroxykaempferol glycosides in Safflower.

The zebrafish offers unique characteristics for studying the pathology and screening therapeutic drugs for cardiovascular diseases. The transparent zebrafish embryo and larvae allows noninvasive observing and imaging the cardiac contraction, and blood flow *in vivo* (Bournele and Beis, 2016). Currently, Safflower, Safflower yellow (SY), and HSYA injections are widely used in treating cerebrovascular and cardiovascular diseases (Zhang et al., 2016). However, little is known about effects of 6-hydroxykaempferol glycosides in cerebrovascular and cardiovascular diseases. The 6-hydroxykaempferol 3,6-*di*-*O*-glucoside-7-*O*-glucuronide (HGG) is a representative 6-hydroxykaempferol glycoside in Safflower. Hence, we examined the effect of HGG on recovering ischemia-reperfusion injury (I/R) and preventing thrombosis. We utilized HUVECs in OGD/R to evaluate the protective effect of HGG on hypoxia/reoxygenation-induced endothelial injury, and revealed its potential molecular mechanisms through RNA-seq analysis. In addition, we used PHZ-induced zebrafish thrombosis model to evaluate the anti-thrombotic effect of HGG *in vivo*.

## Materials and methods

### Experimental materials

The plant material was obtained from Hebei All Thai Pharmaceutical Co., Ltd., (Lot:1711001, Hebei, China), which was identified and authenticated by the authors as the florets of *Carthamus tinctorius* L. Its voucher specimen (NPFFC-2018) was deposited in the Laboratory of Natural Products and Functional Foods, School of Life Sciences, Inner Mongolia University, China. Dulbecco’s modified Eagle medium (DMEM), penicillin-streptomycin (P/S), trypsin-EDTA, and phosphate buffered saline (PBS) were purchased from Gibco (Thermo Fisher Scientific, Suzhou, China). Fetal bovine serum (FBS) was provided by Biological Industries (Kibbutz Beit Haemek, Israel). The 3-(4,5-dimethylthiazol-2-yl)-2,5-diphenyltetrazolium bromide (MTT) was purchased from Sigma-Aldrich (St Louis, Mo, United States). Lactate dehydrogenase (LDH) assay kits were obtained from Nanjing Jiancheng Bioengineering Institute (Nanjing, China). Annexin V-FITC apoptosis kit was purchased from Sungene Biotech (Sungene, China). Hoechest-33342, BCA kit, and antibodies of NF-κB, p-NF-κB, HIF-1α, and β-actin were obtained from Wanleibio (Wanlei, China). NF-κB inhibitor BAY11-7082, and HIF-1α inhibitor Lw6 were purchased from selleck Chemicals (Selleck, Shanghai, China). The IL-6 ELISA Kit was purchased from NEOBIOSCIENCE. The RNAiso Plus, PrimeScriptTM RT reagent kit with gDNA Eraser, and TB Green™ Premix Ex Taq™ II were provided by Takara (Takara, Japan). The chemical constituents of Safflower used in this research were isolated, purified and identified in our laboratory as described previously (Wang et al., 2021).

### Cell culture

HUVECs in DMEM containing 10% FBS, 100 U/ml penicillin, and 100 mg/ml streptomycin were cultured at 37°C in an atmosphere of 5% CO_2_. Culture medium was changed to fresh once a day. At 80% confluence, the cells were passaged with 0.125% trypsin-EDTA solution.

### Oxygen-glucose deprivation followed by reoxygenation model and drug treatment

The experiment was carried out referring to reported method (Lv et al., 2020). Briefly, HUVECs were cultured in a pre-warmed glucose-free DMEM for 12 h. Then, the cells were maintained in hypoxia condition (1% O_2_, 94% N_2_, and 5% CO_2_) for 4 h, and then returned to normoxic condition (95% air, 5% CO_2_) with normal medium for 10 h as reoxygenation. Cao et al. (2022) reported that protocatechualdehyde at 0.72, 1.45, and 3.62 μM significantly improved cell viability after OGD/R injury (*p* < 0.01), and thus HGG was tested at similar concentrations, 0.1, 1, and 10 μM, in this study. To determine the effect of HGG, cells were pre-incubated with HGG for 10 h, followed 4 h of OGD and 10 h of reoxygenation (R) (Lv et al., 2020). The HUVECs with neither HGG nor OGD/R treatment were served as control. At the end of cell treatments, different tests were carried out as described below.

### Cytotoxicity measurements

Cell toxicity and protection effect were measured by three independent assays: MTT, LDH, and Hoechst 33342 staining assays, and apoptosis was assessed by Annexin V-FITC/PI. Briefly, HUVECs were seeded into 96-well plates at a density of 4 × 10^3^ cells per well, and pre-treated with HGG for 10 h, followed 4 h of OGD and 10 h of reoxygenation.

For MTT assay, MTT (0.5 mg/ml, 20 µl) was added to each well, and co-cultured for another 4 h. At the end of incubation, the supernatant was carefully discarded and the formazan product was dissolved in 150 µl dimethyl sulfoxide (DMSO). The optical density was measured with a microplate reader at 570 nm.

LDH release was regarded as an indicator of cell viability and membrane integrity, and measured with LDH assay kit. To visualize the morphological of HUVECs during OGD/R, Hoechst-33342 was carried out. Following the treatment, cells were stained with Hoechest-33342 for 10 min in darkness at room temperature. Afterward, the cells were rinsed twice with PBS and analyzed under fluorescence microscopy.

The percentage of HUVECs apoptosis was analyzed by Annexin V-FITC and propidium iodide (PI). Briefly, the cells were harvested and washed twice with cold PBS. Cell suspension was stained with Annexin V-FITC and PI for 5 min at room temperature in darkness. The apoptotic cell population was analyzed by flow cytometer.

### Library construction for transcriptome sequencing

Following the treatment, total RNA was extracted from HUVECs with RNAiso Plus, and the RNA quantity and purity were assessed using NanoPhotometer^®^ spectrophotometer. Sequencing libraries were constructed as follows: mRNA was enriched with the magnetic-oligo (dT) beads and broken into short pieces by fragmentation buffer, and these fragments were used as templates to synthesize first-strand cDNA with random primers. Second-strand cDNA were synthesized with buffer, dNTPs, DNA polymerase I and RNase. Following end reparation and adenylation of 3′ end tailing, the cDNAs were purified with AMPure XP system. Finally, sequencing adaptors were connected to select different fragments and enrich the final cDNA library. The cDNA quality was assessed on the Agilent Bioanalyzer 2100system.

### Transcription data analysis

Raw data were firstly processed through in-house Perl scripts. During this step, clean data was acquired by removing reads which contain adapter, poly-N, and poor-quality reads from raw data. Simultaneously, Q20, Q30, and GC-content of clean data were calculated. All downstream analyses were performed based on clean data with high-quality. Reference genome files and annotations were directly downloaded from NCBI (https://www.ncbi.nlm.nih.gov/genome/?term = *Homo* + *sapiens*), and then paired-end clean reads were aligned to reference genome with HISAT2. The differentially expressed genes (DEGs) were picked out using the DESeq2 *R* package (1.16.1). For a more in-depth understanding of the screened DEGs, Gene Ontology (GO) function and Kyoto Encyclopedia of Gene and Genome (KEGG) pathway enrichment were performed using clusterProfiler *R* package. *p* < 0.05 was considered as the threshold to determine the DEGs between each treatment of compared samples.

### RT-qPCR

For transcriptome analysis, nine DEGs from RNA-seq were validated by RT-qPCR. cDNA was synthesized from RNA using primeScriptTM RT reagent kit with gDNA Eraser, and RT-qPCR was obtained with TB Green™ Premix Ex Taq™ II. Table 1 shows the primer of randomly selected DEGs and inflammatory markers including interleukin *(IL)-1β*, *IL-6*, and tumor necrosis factor *(TNF)-α*. RT-qPCR amplification was performed on a LightCycler^®^ 480II, and calculated by the 2^−ΔΔCT^ formula.

**TABLE 1:** The primers of RT-qPCR.

Gene	Sequence 5′–3′ fwd. and rev.	Gene	Sequence 5′–3′ fwd. and rev.
*SQSTM1*	TGACCCCCGTCTCTCCA	*CITED2*	AGT​GGT​TGT​GGG​GGT​AGG​GG
	TTCTCATCTGCTCCGCC		GGG​GAC​TGT​GAA​CGG​AGG​GC
*CTIF*	GCCTGCCTCCCCCTTGTC	*GMPR2*	GATGTCTCGGATGGTATG
	TCAGCACCCCACCCCTCA		ACAGTGAGTCAGGTGGTG
*INTS3*	TGG​TAG​GCT​TCG​GTT​TCG​T	*MBD1*	CCC​AGG​AGC​AGG​GAA​TGA​A
	GAT​CTC​TTC​TCC​CTG​GCG​G		GCC​AGC​CAA​GAC​TCG​GAA​A
*NFKBIZ*	GCA​CTG​GCT​GTT​CGT​TCT​C	*JUN*	TGCCTCCAAGTGCCGAAA
	TCA​TTC​GCC​TCT​TTT​TGG​A		GCTGTGCCACCTGTTCCC
*NFKBIA*	GCA​CCC​AAG​GAC​ACC​AAA​AG	*TNF-*α	CCT​CTC​TCT​AAT​CAG​CCC​TCT​G
	AGG​AAA​TAC​CCC​CCT​ACA​CC		GAG​GAC​CTG​GGA​GTA​GAT​GAG
*IL-1β*	AGC​TAC​GAA​TCT​CCG​ACC​AC	*IL-6*	ACT​CAC​CTC​TTC​AGA​ACG​AAT​TG
	CGT​TAT​CCC​ATG​TGT​CGA​AGA​A		CCA​TCT​TTG​GAA​GGT​TCA​GGT​TG
*β-actin*	ACC​CAC​ACT​GTG​CCC​ATC​TA		
	GCC​ACA​GGA​TTC​CAT​ACC​CA		

### Enzyme-linked immunosorbent assay

The level of interleukin-6 (IL-6) in cell supernatants was detected by the corresponding ELISA kits (Enzyme-linked Biotechnology Co., Ltd., Shanghai, China). The optical density was measured using the TECAN microplate reader (tecan Group Ltd., Seestrasse, Switzerland) at 450 nm.

### Western blotting analysis

The protein expression of HUVECs with OGD/R was analyzed by Western blot experiment. HUVECs was collected and lysed in ice-cold RIPA buffer containing protease and phosphatase inhibitors. After BCA assay, equal amounts (40 μg) of protein samples were loaded, separated by 10% SDS-polyacrylamide gel (SDS-PAGE), and transferred to polyvinylidene fluoride membrane. The membrane was blocked with 5% non-fat milk, then incubated with targeted primary antibodies against NF-κB, p-NF-κB, HIF-1α, and anti-β-actin overnight at 4°C. The membrane was washed and then incubated with secondary antibodies IgG-HRP for 1 h at room temperature. Proteins were visualized with ECL reagents and analyzed by Image J.

### Zebrafish thrombosis model and treatment

Adult wild-type AB strain zebrafish were obtained from China Zebrafish Resource Center, Wuhan, China, and the husbandry, breeding and feeding were described previously (Chen et al., 2019; Wang et al., 2021). Thrombosis in zebrafish was induced by PHZ stimulation as described in detail in previous report (Wang et al., 2021), in which, two chemical constituents from Safflower at 10, 50, and 100 μM protected zebrafish against PHZ-induced thrombosis, and 1, 10, 100 μM HGG were tested in this study. Briefly, zebrafish embryos (*n* = 30) were pretreated with HGG (1, 10, and 100 μM) or aspirin (100 μM) (positive control). The zebrafish in control and model groups were treated with DMSO (up to 0.05%). At 72 hpf (hour post-fertilization), the larvae were treated with 3 μM PHZ containing the corresponding concentration of HGG or aspirin, except for controls, and then incubated for 24 h at 28.5 ± 0.5°C. After PHZ treatment, the zebrafish thrombus was qualitatively and quantitatively analyzed by the caudal vein and heart red blood cells (RBC) intensity with O-dianisidine staining. The photo and video data were taken using an Olympus microscope. The anti-thrombotic effect was calculated as following: anti-thrombotic effect (%) = [*S(HGG)*-*S(Model)*]/[*S(Control)*- *S(Model)*] × 100%, where S represents the heart RBC intensity of different treat groups.

## Results

### Cytotoxicity of the chemical constituents of Safflower on human umbilical vein endothelial cells

**FIGURE 1:**
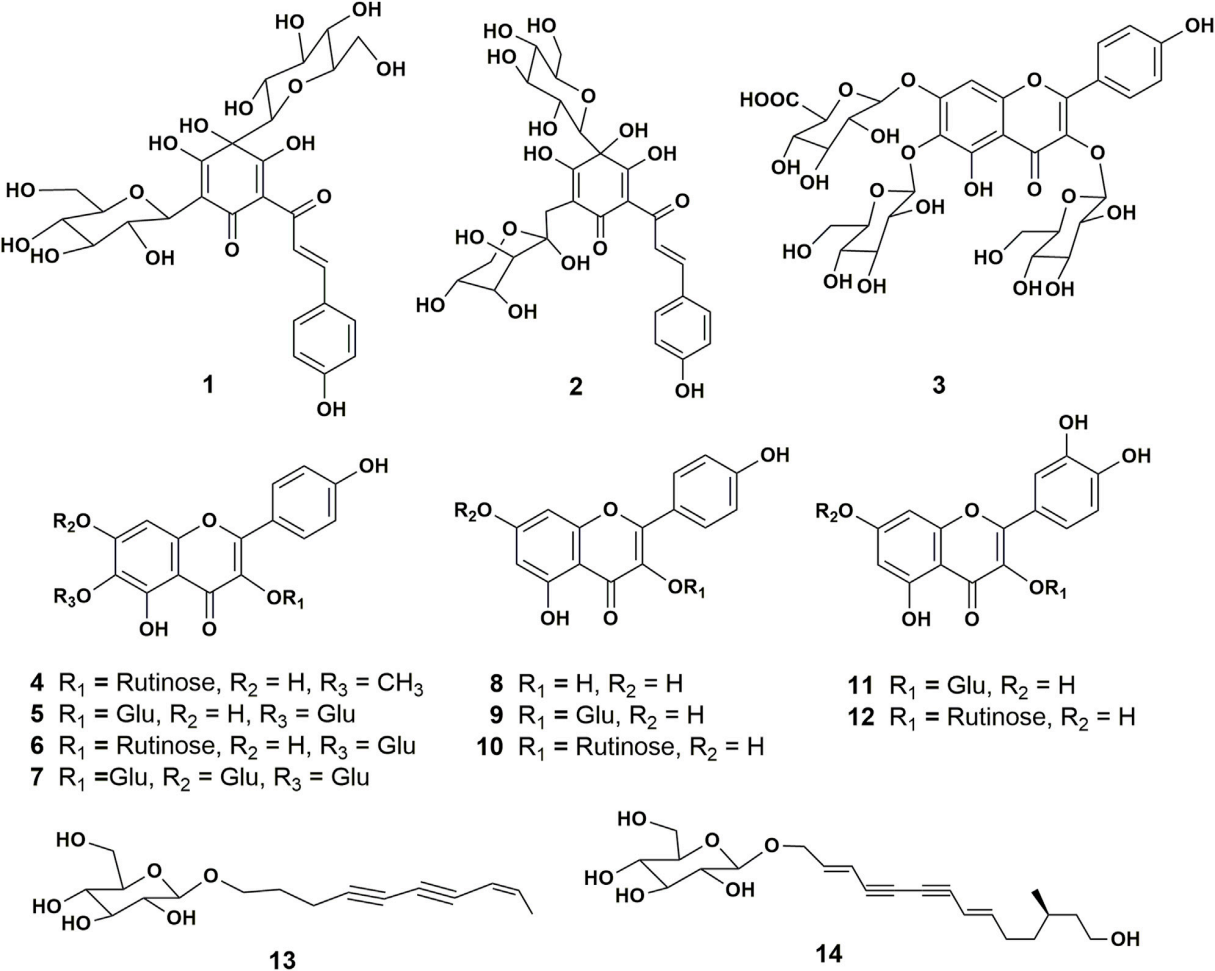
Structures of the chemical constituents isolated and identified from the florets of *C. tinctorius*. **1**, hydroxysafflor yellow A; **2,** hydroxysafflor yellow C; **3,** 6-hydroxykaempferol 3,6-d*i-O*-glucoside-7-*O*-glucuronide (HGG); **4**, 5,7,4’-trihydroxy-6-methoxyflavone-3-*O*-rutinoside; **5**, 6-hydroxykaempferol 3,6-*di-β*-glucoside; **6**, 6-hydroxykaempferol 3-*O-β*-rutinoside-6-*O-β*-glucoside; **7**, 6-hydroxykaempferol 3,6,7-tri-*O-β*-glucoside; **8**, kaempferol; **9**, kaempferol 3-*O-β*-glucoside; **10**, kaempferol 3-rutinoside; **11**, quercetin 3-*O-β*-glucopyranoside; **12**, Rutin; **13**, bidenoside C; **14**, (2E,8E)-tetradecadiene-4,6-diyne-1,12,14-triol-1-*O-β*-glucopyranoside.

**FIGURE 2:**
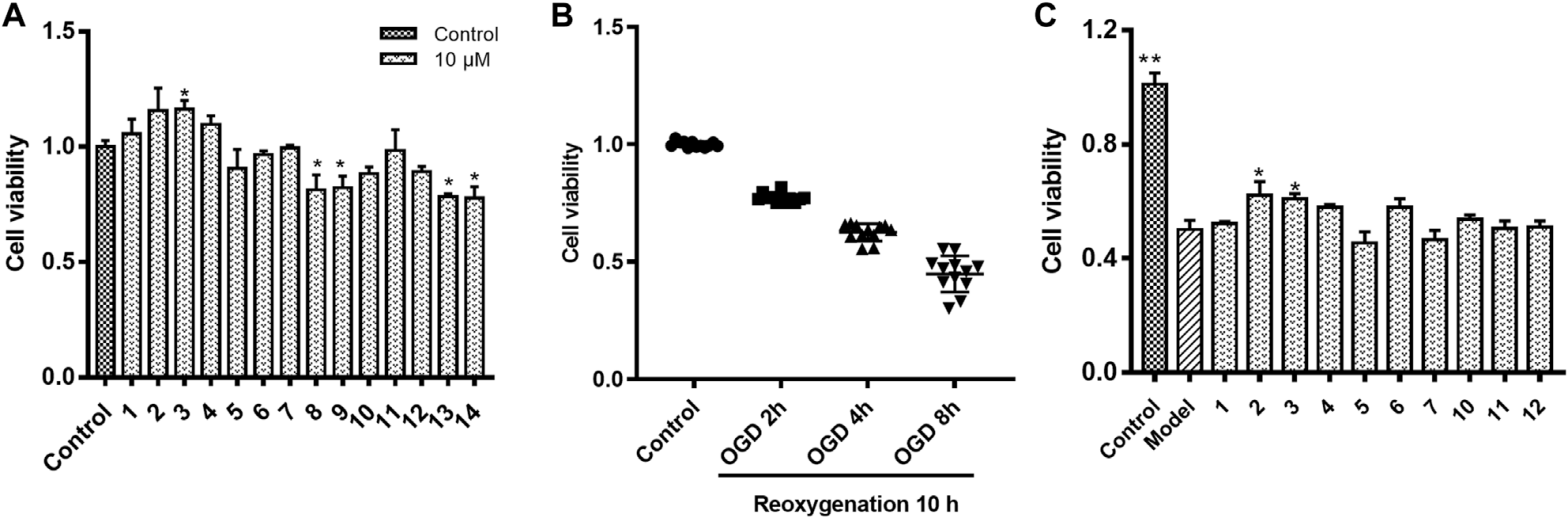
HUVECs viability in different culture compositions. **(A)** HUVECs viability in normoxic condition co-cultured with individual chemical constituent of Safflower; **(B)** HUVECs viability in oxygen-glucose deprivation (OGD) for 2, 4, or 8 h followed by reoxygenation for 10 h; **(C)** Protective effects of compounds 1–7 and 10–12 on HUVECs under OGD/R. *p < 0.05, **p < 0.01 vs. OGD/R.

The cytotoxicity on HUVECs of all the chemical constituents (Figure 1) isolated from Safflower (Wang et al., 2021) were examined by MTT method. As shown in Figure 2A, the chalcones (1, 2), the 6-hydroxykaempferol glycosides (3–7), the quercetin glycosides (11, 12), and the kaempferol diglycoside (10) were non-toxic to HUVECs up to 10 μM, while the kaempferol monoglycosides (8, 9) and the polyacetylenes (13, 14) were toxic to the cells.

### Protective effects of the chemical constituents of Safflower on human umbilical vein endothelial cells under oxygen-glucose deprivation followed by reoxygenation

As shown in Figure 2B, on OGD for 2, 4, and 8 h followed by 10 h reoxygenation, the viability of HUVECs decreased to 77.2%, 62.5%, and 44.8% of control, respectively. On this base, the OGD4/R10 was used to screen the protective effects. Figure 2C shows the protective effects of compounds 1–7, and 10–12 at 0.1 μM on HUVECs under OGD4/R10. Hydroxysafflor yellow C (2) and HGG (3) protected the OGD/R-injured HUVECs significantly (*p* < 0.05). Hydroxysafflor yellow C is a chalcone compound having a similar structure to that of hydroxysafflor yellow A which has been reported to protect hypoxia-induced HUVEC injuries (Lv et al., 2020). HGG represents another characteristic structural type, the 6-hydroxykaempferol glycosides, in Safflower, and its related pharmacologic action has not been reported. Thus, further experiments were carried out on HGG to confirm its effect, and to investigate its underlying mechanisms.

**FIGURE 3:**
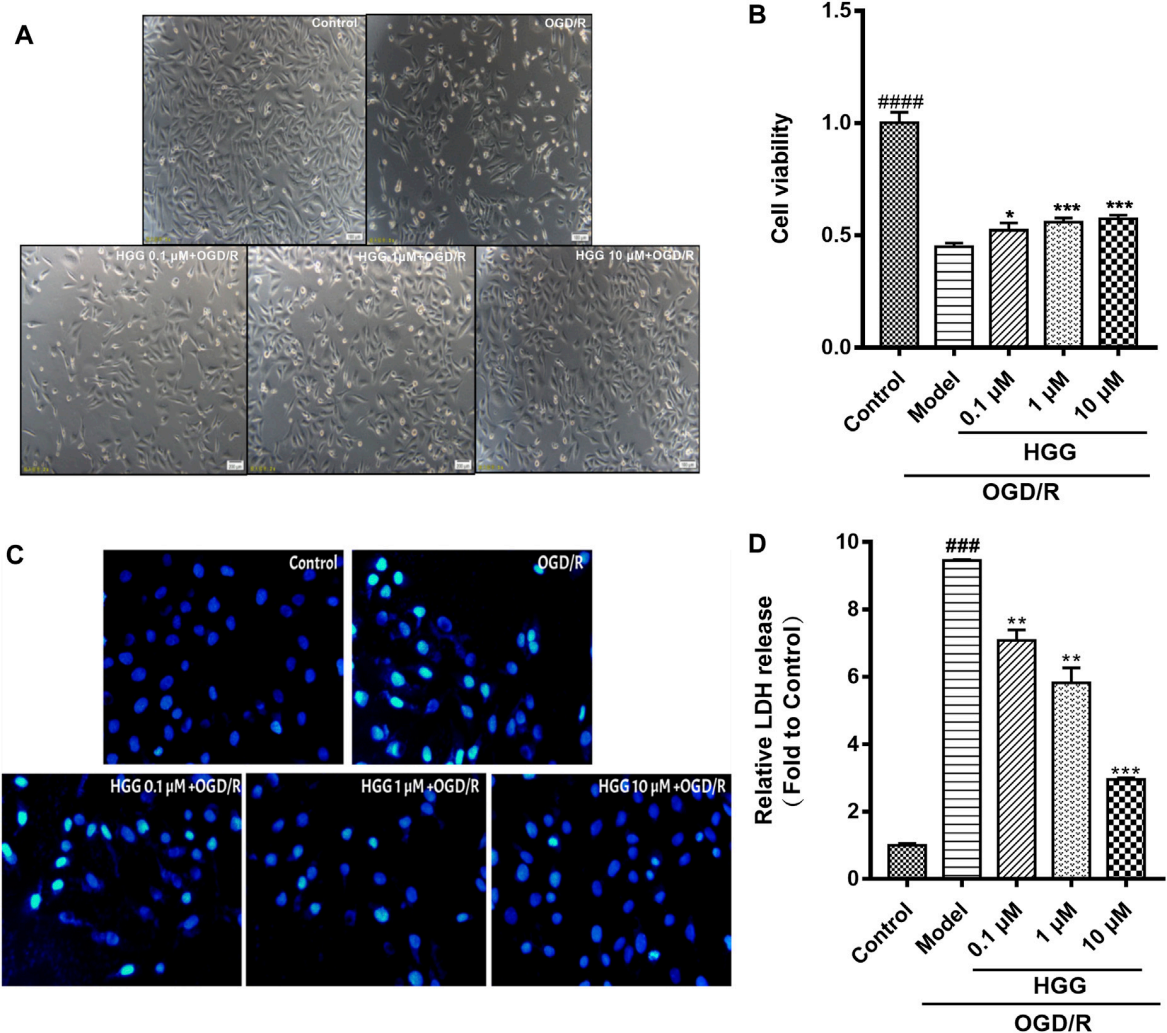
HGG significantly prevents OGD/R-induced damage on HUVECs in a dose-dependent manner. **(A)** Morphology of HUVECs cultured in normoxic, and in HGG-treated HUVECs after OGD/R; **(B)** Cell viability of HUVECs under OGD/R and treated with or without HGG; **(C)** The morphological features of cell apoptosis of OGD/R by Hoechest-33342 staining; **(D)** LDH release in HGG-treated HUVECs after OGD/R; All data are presented as means ± SD from independent experiments performed in triplicate. ^###^ *p <* 0.001, ^####^ *p <* 0.0001 vs. Control; *p < 0.05, **p < 0.01, ***p < 0.001 vs. OGD/R.

**FIGURE 4:**
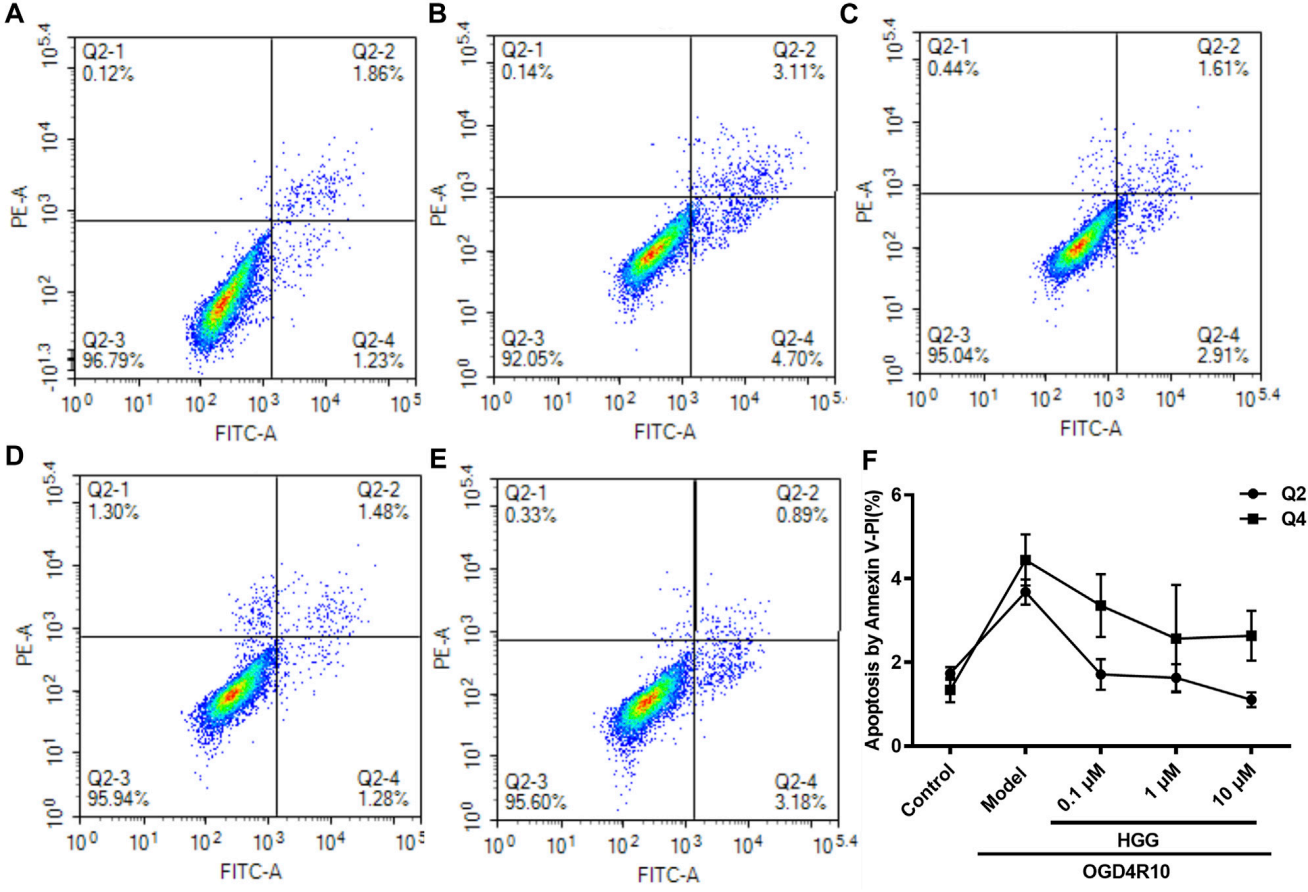
OGD/R-induced apoptosis in HUVECs using Annexin V-FITC/PI staining. **(A)** Control; **(B)** OGD/R; **(C)** 0.1 μM HGG + OGD/R; **(D)** 1 μM HGG + OGD/R; **(E)** 10 μM HGG + OGD/R. Quadrant distinction: upper left corner quadrant for dead cells, lower left corner quadrant for living cells, upper right corner quadrant for advanced apoptotic cells, and lower right corner quadrant for the early apoptotic cells. **(F)** The apoptosis rate of HUVECs after exposure to OGD/R.

The protective effects on HUVECs under OGD/R of HGG at different concentrations (0.1, 1, and 10 µM) were tested. HGG significantly decreased the OGD/R-induced endothelial cell apoptosis in a concentration-dependent manner (Figures 3A,B). As shown in Figure 3C, similar results were obtained by Hoechest-33342 assay, in which apoptotic HUVECs showed strong fluorescence, normal cells displayed weak fluorescence, and necrotic cells have no fluorescence. The results showed that HGG dose-dependently decreased HUVEC apoptosis. LDH is widely used as an indicator of cell apoptosis. The LDH release in ODG/R was 10.7-fold higher than that in the control. Treatment with HGG at 0.1, 1, and 10 µM reduced the LDH release to 8.7-, 6.2-, and 3.8-fold of that in the control, respectively (Figure 3D). Similar results were observed in Annexin V-FITC/PI assay (Figure 4). The apoptotic cells were 3.07% in the control, 7.72% in the OGD/R group, and 5.07%, 4.2%, and 3.74% (*p* < 0.01) in the HGG groups at 0.1, 1, and 10 μM, respectively.

These results strongly indicated that HGG concentration-dependently protected against OGD/R-induced endothelial injury *in vitro.* Consequently, the underlying mechanisms were investigated by RNA-seq and confirmed by Western blot and RT-qPCR.

### Analysis of differentially expressed genes

**FIGURE 5:**
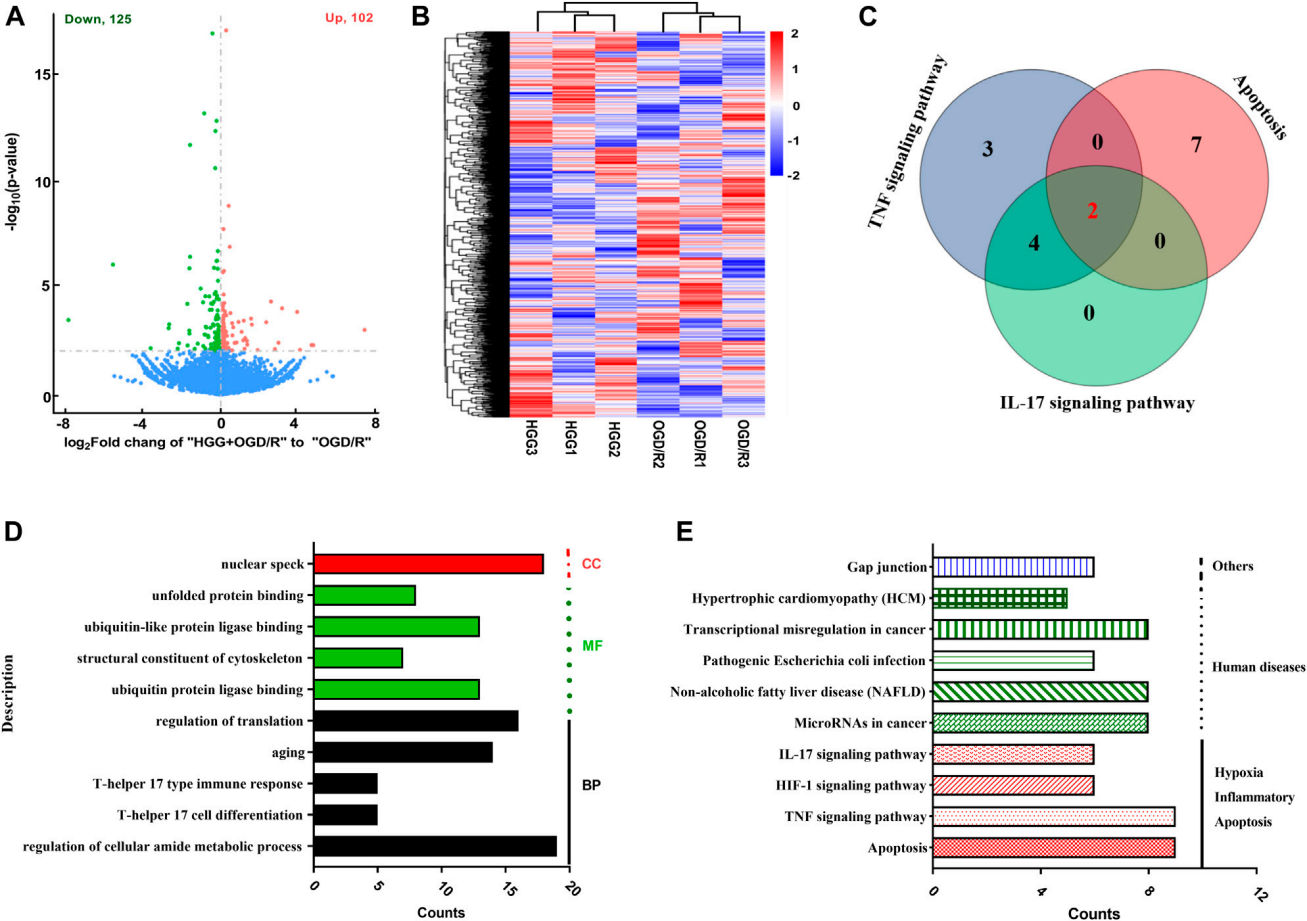
Analysis results from transcription study in OGD/R-exposed HUVECs treated with HGG. **(A)** The volcano graph of 227 DEGs between incubated HGG + OGD/R group and OGD/R group. Red, upregulated DEGs; green, downregulated DEGs; **(B)** Heat map representation; **(C)** The venn diagram of DEGs in KEGG signaling pathway; **(D)** GO enrichment and **(E)** KEGG pathway analysis of 227 DEGs in HGG treated HUVECs after OGD/R.

The high-quality RNA-seq data (Table 2) were used for in depth analysis. For uncovering the potential mechanisms underlying the attenuation of HGG on OGD/R-induced injuries, the 227 DEGs (*p* < 0.05) (Figure 5A; Supplementary Table S1) were subjected to 81 GO (*p* < 0.05) (Figure 5D; Supplementary Table S2) and 212 KEGG pathways (Figure 5E; Supplementary Table S3) analyses. As shown in Figure 5D, the DEGs were classified into 3 functional groups (BP, CC, and MF). DEGs that were mainly enriched in BP group involved in response to decreased oxygen levels, nutrient levels, vascular smooth muscle cell proliferation, inflammatory response, and aging; DEGs in CC group were significantly enriched in nuclear speck; These DEGs in MF group were enriched in the structural constituent of cytoskeleton and protein ligase binding (Supplementary Table S2). According to KEGG enrichment results, we screened out 10 pathways based on the threshold of *p* < 0.01 (Figure 5E), and the results indicated that HGG attenuated OGD/R injury mainly through the TNF, apoptosis, IL-17, and HIF-1 signaling pathways. Venn diagram of DEGs analysis demonstrated that *JUN*, and *NFKBIA* were the hub genes among the apoptosis, TNF, and IL-17 signaling pathways (Figure 5C; Table 3).

**TABLE 2:** Statistical summary analysis of transcriptional sequencing data of HUVECs.

Sample	Means for reads				Means for mapping	
	Raw read	Clean reads	Q20 (%)	GC-pct (%)	Map reads	Unique reads
Control	4,35,09,653	4,29,48,056	97.78	49.39	4,11,89,883 (95.90%)	3,91,33,040 (91.12%)
OGD/R	4,53,41,180	4,46,68,277	97.92	49.18	4,28,27,944 (95.88%)	4,06,98,524 (91.11%)
HGG + OGD/R	5,41,63,926	5,33,47,148	98	49.47	5,11,27,553.5 (95.84%)	4,85,33,437 (90.98%)

**TABLE 3:** DEGs in KEGG signaling pathways.

Signaling pathways	Total	DEGs
Apoptosis, IL-17 and TNF signaling pathway	2	*NFKBIA, JUN*
IL-7 and TNF signaling pathway	4	*CSF2, MMP3, IL6, TNFAIP3*
TNF signaling pathway	3	*SOCS3, LIF, EDN1*
Apoptosis	7	*DDIT3, LMNA, HRAS, TUBA1A, TUBA4A, TUBA1B, LMNB2*

### RT-qPCR validation and Western blot analysis

**FIGURE 6:**
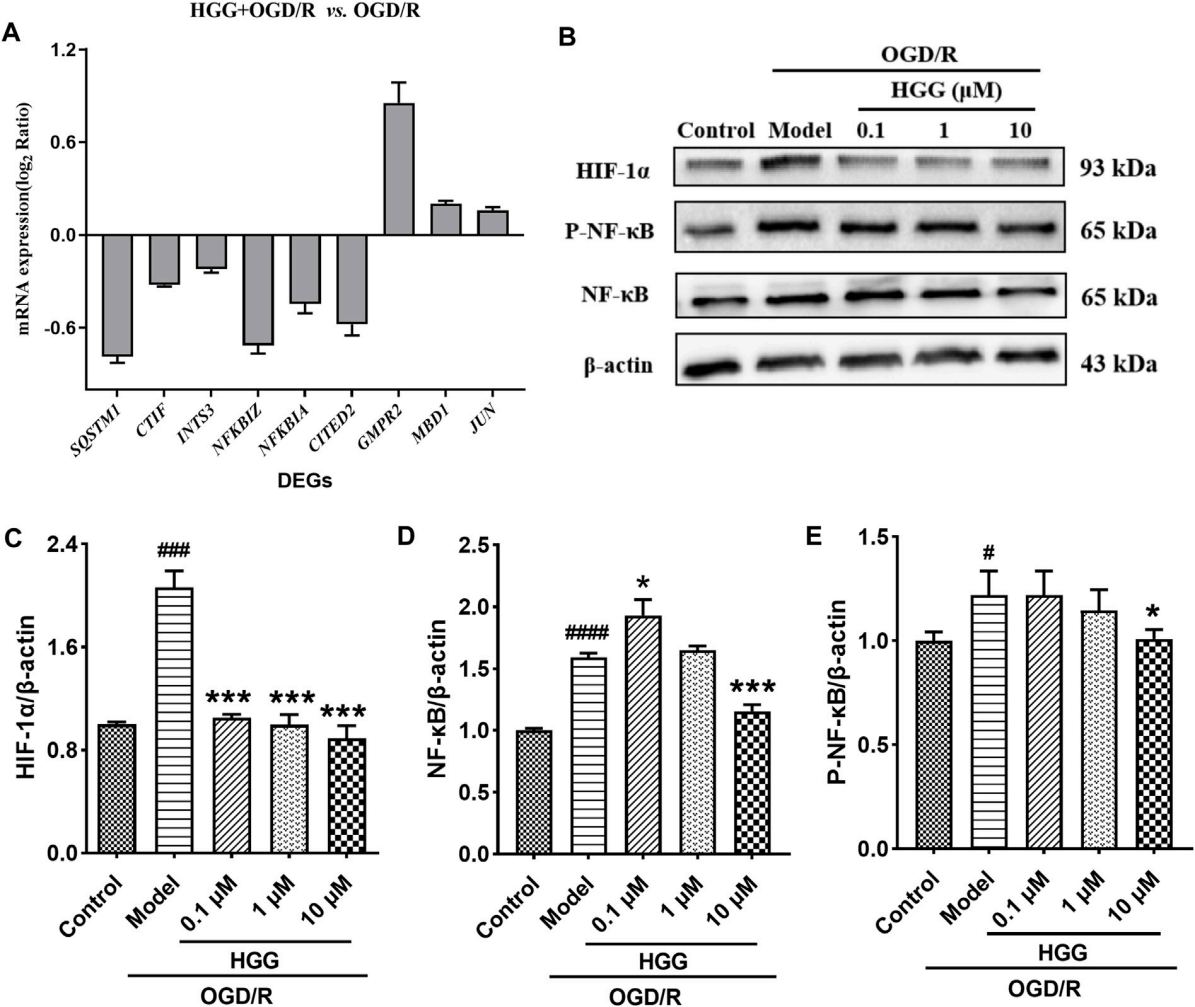
HGG protects HUVECs against OGD/R-induced apoptosis *via* HIF-1α/NF-κB pathway. **(A)** The mRNA expression levels of nine DEGs after OGD/R incubation; **(B)** The protein expression levels of HIF-1α, NF-κB, and p-NF-κB in Western blot experiment for HUVECs under OGD/R and treated with HGG at 0.1, 1, and 10 μM; **(C–E)** Semi-quantification of protein expression by Image J analysis. All data were presented as means ± SD (*n* = 3). ^#^ *p <* 0.05, ^###^ *p <* 0.001 vs. Control; *p < 0.05, **p < 0.01, ***p < 0.001 vs. OGD/R.

To validate reliability of the expression data identified by RNA-seq, nine DEGs were randomly selected for RT-qPCR to determine their relative expression in OGD/R and OGD/R + HGG groups. The results showed similar expression trends between RT-qPCR and RNA-seq (Figure 6A; Table 4), thereby, suggesting that the RNA-seq data were reliable.

**TABLE 4:** Significantly upregulated or downregulated genes in HGG + OGD/R vs. OGD/R.

Gene name	Gene description	log2 FoldChange	*p*-Value	Regulation
*SQSTM1*	Sequestosome 1	−1.69	0.005638	Down
*CTIF*	Cap binding complex dependent translation initiation factor	−1.60	1.64E-06	Down
*INTS3*	Integrator complex subunit 3	−0.85	1.30E-13	Down
*NFKBIZ*	NFKB inhibitor zeta	−0.27	8.40E-13	Down
*NFKBIA*	NFKB inhibitor alpha	−0.19	2.99E-05	Down
*CITED2*	Cbp/p300 interacting transactivator with Glu/Asp rich carboxy-terminal domain 2	−0.43	2.87E-17	Down
*GMPR2*	Guanosine monophosphate reductase 2	2.56	5.43E-05	Up
*MBD1*	Methyl-CpG binding domain protein 1	0.18	6.40E-05	Up
*JUN*	Jun proto-oncogene, AP-1 transcription factor subunit	0.27	2.10E-17	Up

**FIGURE 7:**
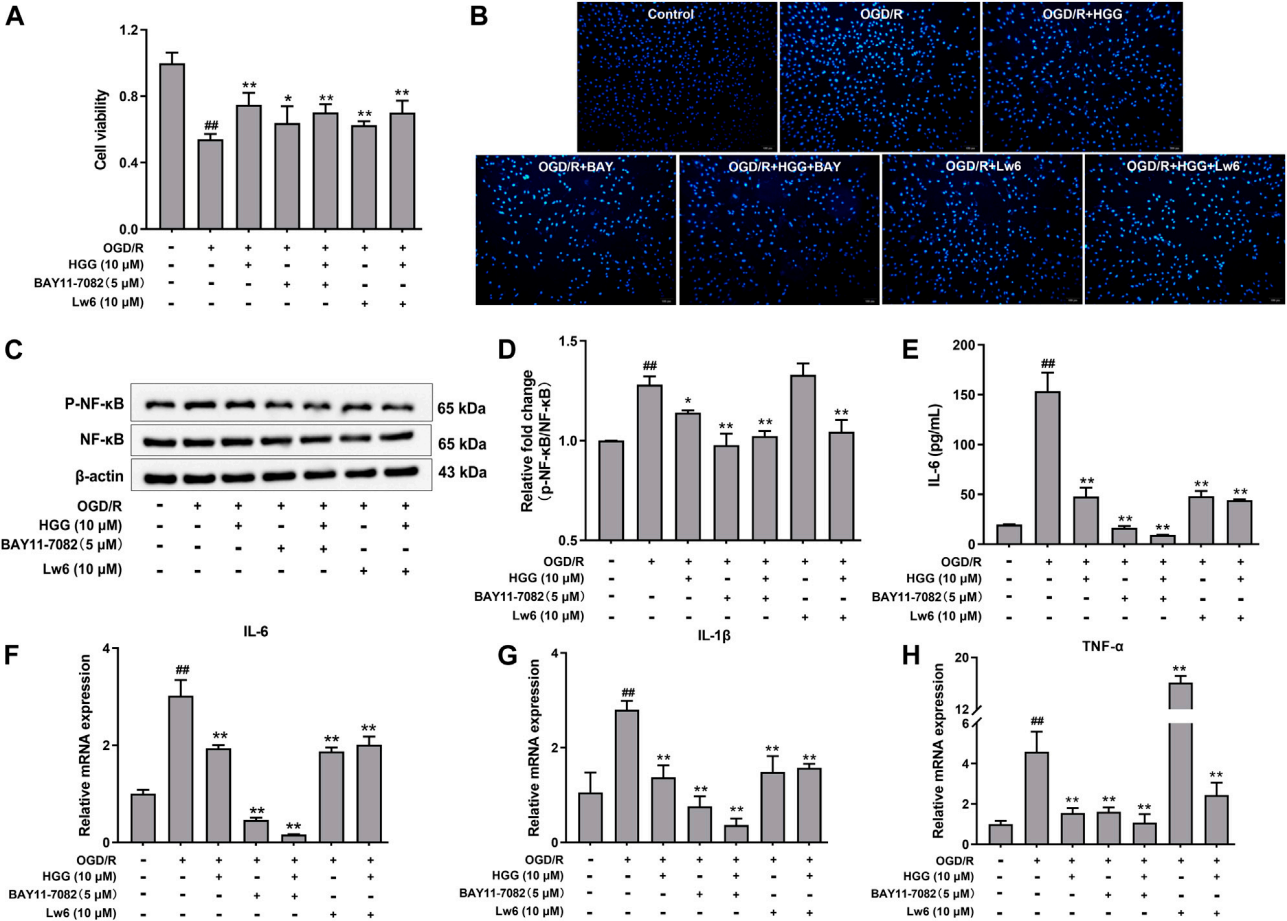
Effects of Lw6 (a HIF-1α inhibitor) and BAY11-7082 (a NFκB inhibitor) on OGD/R induced inflammation in HUVECs. The cell viability, expression of pro-inflammatory cytokines, and Western blot analysis of OGD/R-treated HUVECs in the presence or absence of HGG. **(A)** Cell viability of HUVECs determined by MTT assay. **(B)** Apoptosis assay with hochest-33342 staining, Scale bar = 100 μm. **(C)** The protein expression levels of p-NF-κB and NF-κB were determined using Western blot analysis. **(D)** The relative expression of p-NF-κB/NF-κB in HUVECs after OGD/R. **(E)** Concentration of IL-6 in the cell supernatants was measured by Elisa kits. RT-qPCR was performed to assess the expression of pro-inflammatory cytokines including **(F)** *IL-6*, **(G)** *IL-1β*, and **(H)** *TNF-α*. Data are presented as means ± SD (*n* = 3), ^##^ *p* < 0.01 vs. Control; *p < 0.05, **p < 0.01 vs. OGD/R.

As shown in Figure 6B, upon OGD/R treatment, the protein expression of HIF-1α was upregulated to a level of 2.06-fold higher than that of control (*p <* 0.001). After incubation with 0.1, 1, and 10 μM HGG, the protein expression decreased to 105.0%, 99.7%, and 88.9% (*p* < 0.05) of control. OGD/R treatment also significantly increased the protein expression levels of NF-κB and p-NF-κB as compared to normoxia group, whereas HGG treatment downregulated the expression levels compared to OGD/R group (Figures 6B,D,E). Given that HIF-1α plays a central role in many hypoxic events, we further investigated the protective effects of HGG on the inflammatory response in HUVECs subjected to OGD/R, and the role of the HIF-1α/NFκB signaling pathway in this process. Furthermore, we have used the specific inhibitors Lw6 (10 μM, a HIF-1α inhibitor) and BAY11-7082 (5 μM, a NFκB inhibitor) to verify the mechanism of HGG against OGD/R-mediated inflammatory response. As expected, the protein expression levels of p-NF-κB and NF-κB significantly reduced in OGD/R-stimulated HUVECs after treatment with BAY (Figures 7C,D), confirmed the central role of NFκB signaling pathway in OGD/R-induced endothelial dysfunction. Compared with OGD/R group, inhibitors Lw6 and BAY significantly alleviated endothelial injuries (Figures 7A,B), mitigated OGD/R-induced increases in inflammatory markers including *IL-6*, *TNF-α* and *IL-1β* mRNA expression (Figures 7F–H), and inhibited the secretion of pro-inflammatory cytokine IL-6 (Figure 7E). OGD/R promoted the mRNA expression of pro-inflammatory cytokines, such as *IL-1β*, *IL-6*, and *TNF-α*, and resulted in production of pro-inflammatory cytokine IL-6 in the culture supernatant, while pretreatment with HGG inhibited *IL-1β*, *IL-6*, and *TNF-α* mRNA expression and the release of IL-6 (Figures 7E–H). These results confirmed that HGG prevented HUVECs from OGD/R-induced injury partly through the regulation of HIF-1α/NF-κB pathway, and that HGG may have therapeutic value for protecting endothelial cells from IR injury.

### 6-hydroxykaempferol 3,6-di-O-glucoside-7-*O*-glucuronide mitigated phenylhydrazine-induced thrombosis

**FIGURE 8:**
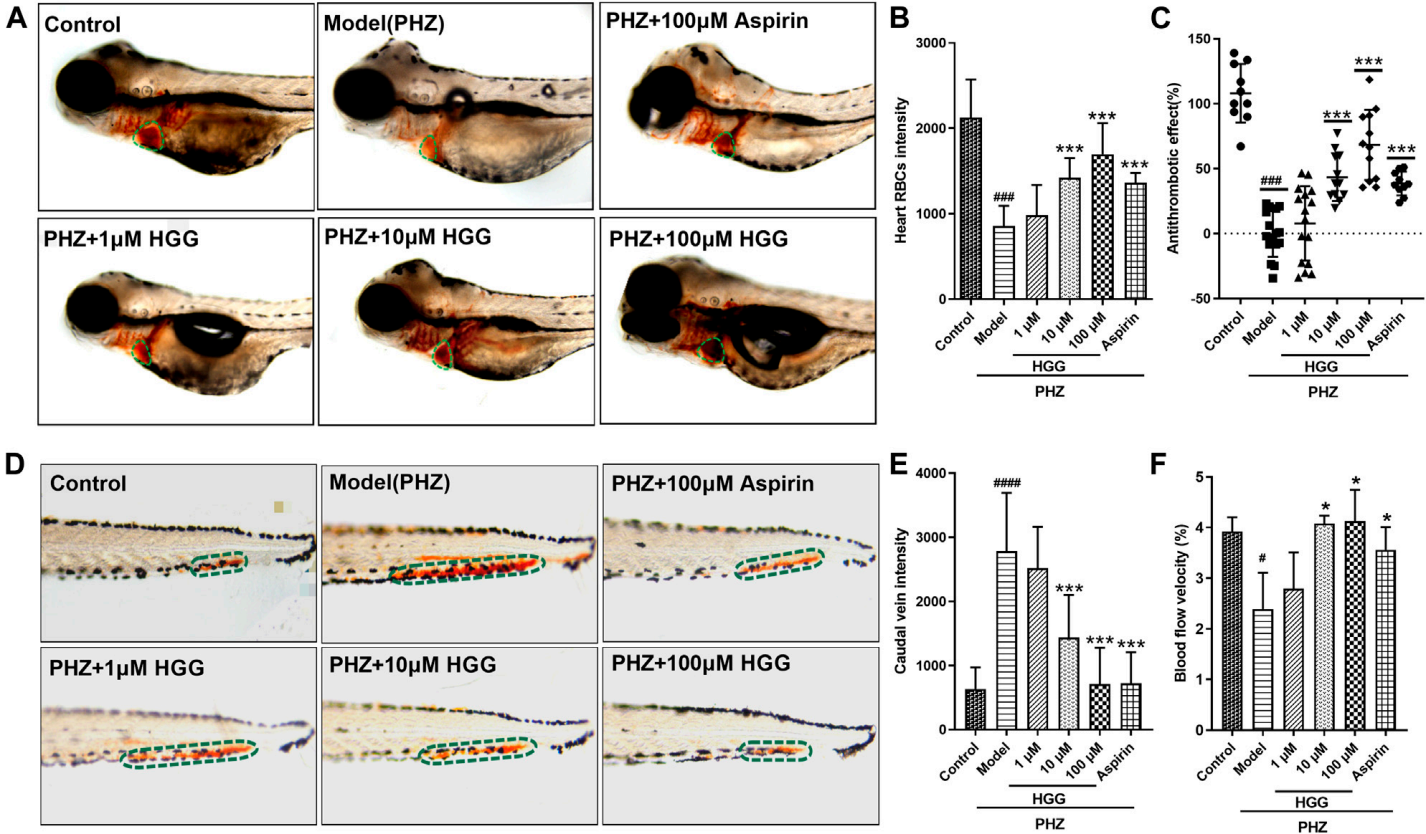
Thrombus formation and hemodynamic changes in PHZ-stimulated zebrafish with different concentrations of HGG, and anti-thrombotic drug aspirin. **(A)** Representative images of the heart RBC (green dotted line) stained with O-dianisidine after HGG, or aspirin treatment; **(B)** Quantitative analysis of heart RBCs intensity; **(C)** The anti-thrombotic effect based on heart RBC intensity; **(D)** Erythrocytes aggregation in the caudal vein (circled with green dotted line); **(E)** Quantitative analysis of the caudal vein intensity for thrombotic zebrafish; **(F)** Determination of blood flow in the caudal vein after treated with PHZ, and co-treated with HGG. ^###^ *p < 0.001* vs. Control, ***p < 0.001 vs. Model.

To evaluate the protective effect of HGG on PHZ-induced zebrafish thrombosis model, the O-dianisidine stained zebrafish images were captured by microscope. After PHZ treatment, the red blood cells (RBCs) initiated to aggregate and accumulate in the caudal veins, suggesting thrombus formation (Figures 8D,E; Supplementary Video S1). Notably, compared with OGD/R group, pre-treatment with HGG at 1, 10, 100 µM significantly enhanced zebrafish heart RBCs intensity to a level similar to that treated with 100 µM aspirin (positive control) (Figure 8D), indicating that thrombosis formation was reduced by HGG. The anti-thrombotic effect was 33.0% for 100 μM aspirin, and 11.9%, 43.3%, and 64.0% for 1, 10, and 100 µM HGG, respectively (Figure 8A,C; Supplementary Video S2). The results also showed for the first time that HGG significantly inhibited the tail vein thrombus and restored the quantity of heart RBCs in zebrafish thrombosis model. We subsequently examined the effects of HGG on the blood flow rate of zebrafish tail vein at selected regions. Compared with OGD/R group, 10, 100 µM and HGG treatment significantly increased the blood flow rate to the level similar to that treated with 100 µM aspirin (Figure 8F). These results indicate that HGG could significantly inhibit PHZ-induced thrombosis *in vivo*.

## Discussion and conclusion

The objective of this study was to investigate the protective effects and possible mechanism of HGG from Safflower on human umbilical vein endothelial cells (HUVECs) subjected to oxygen-glucose deprivation/reoxygenation (OGD/R) *in vitro,* and evaluate its anti-thrombotic effects in PHZ-induced zebrafish thrombosis *in vivo*. Excitingly, treatment with HGG successfully alleviated OGD/R-induced endothelial dysfunction. To gain further insight into the potential mechanisms, an integrative analysis of transcriptome data under OGD/R and HGG + OGD/R treatment in HUVECs was performed. According to GO and KEGG analysis, these DEGs were mainly involved and enriched in apoptosis and inflammatory pathways. HGG reduced OGD/R-induced cytotoxicity and apoptosis. HGG protected OGD/R injury by suppressing inflammatory response *via* HIF-1α/NFκB signaling pathway in HUVECs. Furthermore, HGG exhibited anti-thrombotic effects in PHZ-induced zebrafish thrombosis model, enhanced the blood flow rates, restored the heart RBCs intensity and alleviated caudal vein thrombus *in vivo*. Therefore, HGG exerted protective effects on endothelial dysfunction by HIF-1α/NF-κB pathway and prevented from PHZ-stimulated zebrafish thrombosis by ameliorating vascular obstruction and enhancing the blood flow.

AMI is the leading cause of hospitalizations and deaths worldwide (Collaborators et al., 2016). AMI is usually caused by thrombotic occlusion of coronary artery and atherosclerotic plaque rupture or erosion of endothelium, leading to inadequate of oxygen and nutrients, which is characterized by endothelial injury and thrombus formation (Reed et al., 2017; Lv et al., 2020). Timely reperfusion therapy after AMI has been considered the most effective treatment for reducing acute myocardial injury, protecting heart function, and improving clinical outcomes. However, to some extent, reperfusion itself has been regarded as a “double-edged sword”, as reperfusion may induce cell apoptosis and aggravation of myocardial damage, a phenomenon termed ischemia-reperfusion (I/R) injury (Yellon and Hausenloy, 2007; Neri et al., 2017). I/R injury is a complicated pathophysiological process involving cardiomyocyte apoptosis, endothelial dysfunction, and inflammatory reaction (Cao et al., 2020; Wu et al., 2020). Among them, endothelial dysfunction, an important characteristic of I/R, plays an important role in the development of atherosclerosis and thrombus formation (Heusch, 2019). Thus, alleviation of endothelial injury has become a strategy to prevent and treat acute myocardial infarction.

Endothelial cells form the first barrier between the circulating blood and the surrounding tissues, and the integrity of vascular endothelium has a crucial role for the maintenance of vascular homeostasis. In turn, endothelial injury has been considered an early marker of vascular complications, such as atherosclerosis and thrombosis (Wu et al., 2019). A considerable body of evidence has demonstrated that the endothelium is sensitive to ischemia-reperfusion (I/R) or hypoxia-reoxygenation injury, and the maintenance and repair of vascular endothelial cells determines recovery from injury (Somani et al., 2019). To assess the effect of HGG on I/R injury *in vitro*, we used an OGD/R model in HUVECs by treating them with OGD/R-induced endothelial injury *in vitro* to mimic I/R injury. Apoptosis, a form of programmed cell death that is activated under hypoxic stress is a major cause of I/R injury. Therefore, inhibiting HUVECs apoptosis during OGD/R-injured may effectively ameliorate the myocardial infarction during I/R injury (Yu et al., 2016). In this study, we demonstrated that HGG alleviate endothelial injury and significantly attenuate OGD/R-injured apoptosis of HUVECs *in vitro*. Moreover, we identified several DEGs with enriched apoptosis and inflammatory pathways underlying OGD/R injury by RNA-seq coupled with bioinformatic analysis. Inflammatory response to I/R injury plays a pivotal role in acute myocardial infarction, making it a potential therapeutic target (Ong et al., 2018). Following I/R injury, the initial pro-inflammatory response is activated, and a large number of pro-inflammatory cytokines, such as TNF-α, IL-6, IL-1β, promote the inflammatory response’s progression (Zhao et al., 2000; van Hout et al., 2016). Our results showed that HGG could inhibit the mRNA expression of pro-inflammatory factors *TNF-α*, *IL-6*, and *IL-1β* and regulate the levels of IL-6 in culture supernatant of HUVECs with OGD/R injury. NF-κB as an important transcription factor plays an important role in I/R injury involved in inflammation, apoptosis, and oxidative stress (Liu et al., 2017). In I/R injury, activated NF-κB transfers to the nucleus, and thereby participating in I/R injury. Matsui et al. (2005) found that inhibition of NF-κB activation reduced I/R injury in rats. Similarly, we also found that HGG significantly attenuated OGD/R-induced HUVECs injury and decreased the protein expression of NF-κB.

I/R injury may initially provoke hypoxia response. Insufficient oxygen supplement may lead to aggravation of I/R injury and AMI (Wu et al., 2018). Hypoxia inducible factor 1α (HIF-1α) is a master transcription factor that responses to hypoxia or reduction of available oxygen. In normoxia, the HIF-1α is unstable and easily degraded by prolyl hydroxylases. However, under hypoxia, the degradation and accumulation of HIF-1α is inhibited (Semenza, 2007). Yu et al. (2020) found that inhibition of serum HIF-1 and VEGF concentrations can improve the left-ventricular function in rats with AMI. In this study, we found that the HIF-1α protein in HUVECs suffered with OGD/R injury significantly increased, whereas HGG treatment ameliorated such phenomenon.

AMI usually occurs when ruptured plaques of atherosclerotic cause artery occlusion and thrombosis and unruptured plaques lead to severe coronary stenosis, reducing blood supply to heart region, and leading to irreversible myocardial ischemic injury (Libby, 2013). Therefore, anti-thrombotic therapy is required to stabilize vulnerable plaques and eliminate occlusion to restore vessel patency, reduce I/R injury, and improve cardiac function in patients with AMI (Kumar and Cannon, 2009). Safflower is frequently used in Chinese medicinal formulae for cerebrovascular and cardiovascular diseases (Chinese Pharmacopoeia Committee, 2020). To assess the anti-thrombotic effect of HGG *in vivo*, we tested the compound in a zebrafish thrombosis model. Aspirin, as a clinically anti-platelet drug, is extensively used to treat cardiovascular diseases. Aspirin could decrease the blood viscosity and ameliorate artery vascular stenosis or obstruction, exerting its protective effect in cardiovascular and cerebrovascular diseases (Cortellini et al., 2017). In this study, we demonstrated that the 6-hydroxykaempferol glycoside of Safflower, HGG, had potent anti-thrombosis activity similar to aspirin. Both HGG and aspirin significantly enhanced blood flow velocity and alleviated zebrafish tail vascular stenosis or occlusion.

In conclusion, 6-hydroxykaempferol-3,6-*di*-*O-β*-glucoside-7-*O-β*-glucuronide (HGG), as a major 6-hydroxykaempferol glycoside in Safflower protects OGD/R-induced ischemia-reperfusion injury *in vitro*. The mechanism of action involves the mitigation of inflammatory response through HIF-1α/NF-κB signaling pathway. In addition, HGG has anti-thrombotic activity on PHZ-induced thrombosis in zebrafish and enhances blood flow and alleviates vascular occlusion. To sum up, HGG or Safflower may protect against ischemia/reperfusion injury and ameliorate thrombus formation.

## Data Availability

The original data of this study are included in the article or Supplementary Material. Further inquiries can be directed to the corresponding authors.
